# Effectiveness and Feasibility of Blood Flow Restriction Training for People with Multiple Sclerosis: A Systematic Review

**DOI:** 10.3390/neurolint16060104

**Published:** 2024-11-07

**Authors:** Aitor Blázquez-Fernández, Selena Marcos-Antón, Roberto Cano-de-la-Cuerda

**Affiliations:** 1 International PhD School, Rey Juan Carlos University, 28008 Madrid, Spain; aitorblazquezfernandez@outlook.es; 2 Asociación de Leganés de Esclerosis Múltiple (ALEM), Leganés, 28915 Madrid, Spain; 3 Department of Health Sciences, Universidad Villanueva, 28034 Madrid, Spain; 4 Department of Physical Therapy, Occupational Therapy, Physical Medicine and Rehabilitation, Faculty of Health Sciences, Rey Juan Carlos University, Alcorcón, 28922 Madrid, Spain; roberto.cano@urjc.es

**Keywords:** multiple sclerosis, exercise, physical activity, blood flow restriction training, strength, resistance

## Abstract

Background: Multiple sclerosis (MS) is an immune-mediated inflammatory disease that primarily targets the myelin of axons. Extremities are frequently affected, resulting in a negative impact on both activities of daily living (ADL) and quality of life. In recent years, there has been increasing interest in the potential benefits of exercise and blood flow restriction training (BFRT) programs as a therapeutic tool in people with neurological disorders. The aim of the present systematic review was to know the clinical effects of BFRT programs in people with MS. Methods: A systematically comprehensive literature search was conducted and registered in PROSPERO prior to its execution under the reference number CRD42024588963. The following data sources were used: Pubmed, Scopus, Web of Science (WOS) and the Cochrane Library. The following data were extracted from the papers: study design, sample, interventions, dosage, outcome measures and results. To assess the methodological quality of the papers included, the Quality Index of Downs and Black was used. Additionally, the articles were classified according to the levels of evidence and grades of recommendation for diagnosis studies established by the Oxford Center for Evidence-Based Medicine. Also, the Cochrane Handbook for Systematic Reviews of Interventions was used by two independent reviewers to assess risk of bias, assessing the six different domains. Results: Seven articles with a total of 71 participants were included in the review. Of the seven articles, five papers studied the effectiveness of BFRT combined with strengthening exercises and two papers studied the effect of BFRT combined with aerobic exercise. Of the five articles that analyzed BFRT combined with strengthening exercises, only two presented a control group. Both performed a low-load resistance training in combination with BFRT with four series, 30/15/15/15 repetitions and a rest of 1 min between the series and 3 min between the exercises. The control groups to which they were compared performed a high intensity strengthening exercise protocol which had the same exercises, sets, rests and duration of the protocol as the experimental groups. For those two papers which investigated the effects of BFRT combined with aerobic training, exercise was performed in two sessions per week for a period of 8 and 6 weeks, respectively. In both studies, the experimental protocol began with a warm-up phase and ended with a cool-down phase, and there were differences in cuff management. All these investigations found positive effects in the interventions that combined exercise with BFRT. The characteristics, outcome measures, effects of the interventions and the assessment of the methodological quality of the included studies and risk of bias are shown in the tables. Conclusions: BFRT in people with MS appears to be effective and safe for people with MS. BFRT might show positive clinical effects on strength, hypertrophy and balance outcomes. Nevertheless, future research should be conducted with better methodological quality to ensure the potential benefits of BFRT in people with MS since the studies analyzed present a high risk of bias and methodological limitations.

## 1. Introduction

Multiple sclerosis (MS) is an immune-mediated inflammatory disease that primarily targets the myelin of axons in the central nervous system (CNS), leading to varying degrees of damage to both the myelin and axons [1]. In addition to this, the disease may also result in gliosis and neuronal loss. To date, the exact cause of the disease remains unknown, though it may be linked to a hereditary predisposition combined with an environmental trigger [2]. The main risk factors identified are as follows: (a) low vitamin D levels; (b) genetics and family history, as genetic studies have shown a connection between first, second and third degree relatives; (c) infections, as bacterial or viral infections may favor the subsequent development of MS in genetically susceptible individuals; (d) injuries, as severe injuries directly damaging the brain or spinal cord have been investigated as possible triggers for MS; (e) smoking, as smokers with MS have a worse long-term prognosis and a higher incidence of brain atrophy than those who are non-smokers [1].

Epidemiologically, MS is the leading cause of non-traumatic disability in young adults in Europe and North America [1,2]. On the other hand, the African continent and countries such as Mexico, Brazil, Peru and Venezuela show a prevalence of <10/100,000 people with MS. Australia shows a prevalence ranging from 10–30/100,000 persons with MS to 30–90/100,000 persons with MS. The prevalence in the Asian continent, particularly in its eastern part, is difficult to determine due to the lack of data from different countries [2]. Although in the 1990s the prevalence was equal for both genders, today, particularly in industrialized countries, the prevalence is 3:1 (women/men) [1]. The disease can develop at any age, but its presentation is mostly located between the second and fourth decade of life [1,3]. The cost of the disease includes both medical and non-medical and direct and indirect costs [4]. In Europe, the annual expenditure is estimated to be EUR 15.5 billion per year, around EUR 37,000 per person, while in the United States the figure is around USD 52,000. The average annual expenditure for MS is higher than for most neurological diseases according to the European Brain Council [4]. To date, there is no pharmacological treatment available to cure the disease; therefore, the aim of interventions for MS is focused on reducing subclinical disease activity, preserving maximum brain volume and slowing or preventing the development of disability. Different disease-modifying treatments have been effectively developed for people with relapsing remitting MS. These drugs have been shown to have an effect on the development of the disease, decreasing relapses, presenting a certain halt in the progression of disability and reducing the number of new lesions or effects on brain atrophy. It seems important to point out the role of statin drugs which have shown a promising effect on disability progression, which could support the immunomodulatory and neuroprotective role and serve as a basis for future randomized controlled trials that apply statins as a monotherapy or in combination with other established therapies for the EM. Although these drugs seem to have a certain positive effect in recurrent remitting courses, they fail in progressive courses [4]. Hence, there is a need for other types of complementary therapeutic approaches.

MS affects most of the spheres of life, both of patients and their socio-familial environment. Patients with MS may present physical, cognitive and emotional impairment, resulting in decreased physical activity [5,6]. Extremities are frequently affected, with weakness, spasticity, impaired coordination, tremor, fatigue or dexterity deficits being typical [7,8], resulting in a negative impact on both activities of daily living (ADL) and quality of life [9]. The complexity of the disease, the difficulty in determining the appropriate drug and the combination of symptoms that can occur result in the need for a multidisciplinary approach, including pharmacological, rehabilitative and social therapy [10,11].

In recent years, there has been increasing interest in the potential benefits of exercise as a therapeutic tool to address the consequences of MS [12,13]. To date, the main effects of physical exercise that have been observed have been improvements in physiological capacity, symptomatological level and quality of life. According to scientific evidence, physical exercise is an effective and safe strategy for the treatment of certain MS symptoms [12,13,14]. Recommendations for the application of aerobic exercise in people with MS indicate a weekly frequency of two to five sessions according to the patient’s tolerance, preferably on days when no strength work is developed, with an intensity of 40–70% of VO_2_max, 60–80% of maximum heart rate or a score of 11–13 on the rate of perceived exertion (RPE) scale. Depending on the subject’s level of involvement, the duration of exercise can range from 10 to 40 min, or the exercise can be a 30 min session divided into three sets of 10 min for subjects not adapted to exercise. During the first 2–6 months, progression is recommended to increase the duration or frequency of the training; after these 6 months, and having verified that the intensity is well tolerated by the patient, moderate-intensity training can be replaced by high-intensity intervallic training (>90% VO_2_max) [12]. On the other hand, the review carried out by Kim et al. [13] provides a series of general indications for traditional strength training, highlighting the following: a frequency of two to three sessions per week, starting with two sessions per week and progressing to three over time; between five and ten exercises per session, with one to three sets per exercise; between eight and fifteen repetitions; and rests ranging from 2 to 4 min between sets. Exercises should focus on large muscle groups and include in these patterns those muscles that are weaker or present a greater degree of functional deficit. At the same time, the variability of symptoms present in people with MS should be considered for the correct prescription of exercise, especially considering fatigue and sensitivity to heat.

Regarding strengthening exercise, the traditionally most effective formula is *high-load resistance training* (HL-RT) which involves a load of 70–85% of one-repetition maximum (1RM), described by the American College of Sports Medicine (ACSM) [15]. However, this type of training may not be tolerated by all people with MS, so it is of interest to study various forms of strengthening using different percentages of 1RM, such as *low-load resistance training* (LL-RT) that applies loads at around 20–50% of 1RM [15].

In this context, blood flow restriction training (BFRT) is a training modality performed using concurrent limb occlusion, partially restricting arterial inflow and the venous return of blood from the working muscles. This type of training is typically, but not always, performed in parallel to LL-RT, with repetitions close to muscle failure in each set [16]. However, it can also be performed in parallel to functional or task-focused training, such as horizontal walking, stationary cycling or movements like those present in ADLs. BFRT is achieved by applying an external pneumatic tourniquet at the proximal end of the upper or lower extremity, applying an occlusion pressure promoting ischemia and blood pooling in the capillary beds of the musculature distal to the tourniquet. The combination of ischemia and mechanical tension during the exercise creates an environment of mechanotransducive and metabolic stress generating greater neuromuscular and hormonal adaptations than LL-RT alone and leading to improvements in muscle hypertrophy and strength [16,17]. In the field of physiotherapy, BFRT has become increasingly important since it has awakened interest in its application as a complement to therapeutic exercise, particularly in those subjects who cannot tolerate high loads due to clinical considerations. The systematic review conducted by Reina-Ruiz et al. [17] analyzed the effects of the use of the BFRT system in combination with LL-RT and aerobic exercise in people with different pathologies (anterior cruciate ligament reconstruction, stage two of chronic kidney disease, coronary heart disease, end-stage renal disease, elderly coma patients, knee osteoarthritis and recurrent nonspecific low back pain). The results obtained in terms of strength, despite the heterogeneity of the outcome measures used, present the BFRT system as an effective tool for strength improvement, being equal or superior to HL-RT. Vinilo-Gil et al. [18] conducted a systematic review about the use of the BFRT system in populations with different neurological pathologies (spinal cord injury, stroke and cerebral palsy). Their results showed improvements in sensorimotor function, frequency, gait length symmetry, gait speed and muscle thickness. However, no improvements in balance were found. The systematic review conducted by Jønsson et al. [19] reported that BFRT generated higher strength improvements in people with intrusion body myositis compared to those who did not undergo any type of intervention and in subjects with incomplete spinal cord injury who performed the BFRT protocol in combination with functional electrical stimulation (FES), showing greater muscle circumference in the quadriceps than those who only received FES-based treatment, with unclear results in people with MS.

Therefore, and considering the high prevalence of strength impairments in people with MS, the aim of the present systematic review was to know the clinical effects, the possible presence of side-effects and the therapeutic feasibility of BFRT programs in people with MS.

## 2. Methods

### 2.1. Design

The Preferred Reporting Items for Systematic Reviews and Meta-Analyses (PRISMA) [20] was used to carry out this systematic review, starting with a PICO (patient/population, intervention, comparison and outcome) question: What are the clinical effects of BFRT in people with MS that improve motor control compared to other interventions or no intervention whatsoever? This systematic review was registered in PROSPERO prior to its execution under the reference number CRD42024588963.

### 2.2. Search Strategy

A systematically comprehensive literature search was conducted from January to August 2024 in order to identify original studies that answered the PICO question, using the following data sources: Pubmed, Scopus, Web of Science (WOS) and the Cochrane Library. After identifying eligible articles, a cross-search of their references was also completed for additional studies (Table 1).

The combinations of keywords were as follows: (“blood flow restriction” OR “blood flow restricted” OR “blood flow restriction exercise” OR “blood flow restriction training” OR “blood flow restriction therapy” OR “occlusion training” OR “kaatsu”) AND (“rehabilitation” OR “muscle strength” OR “upper limb” OR “lower limb” OR “resistance training”) AND (“multiple sclerosis”). The detailed search strategy for each database is shown in Table 2.

Two authors independently searched and screened the titles and abstracts to identify studies meeting the inclusion criteria. Duplicates were removed and disagreements regarding the selection of studies were resolved by a third author.

### 2.3. Study Selection

Studies published in English without a restriction of year were considered for inclusion in this review, regardless of their methodological design. The exclusion criteria were as follows: studies not related to MS, no access to the full text, poster communications, congress or symposium reports, technical analysis studies with no clinical application or perspective and studies that included only healthy subjects.

### 2.4. Participants

This review only considered studies that included people with MS without taking into consideration their Expanded Disability Status Scale (EDSS) or their type of MS. The following inclusion criteria were established: (a) patients with a diagnosis of MS; (b) people of both male and female genders; (c) the use of the BFRT program; and (d) papers published independently of their year and language of publication.

### 2.5. Interventions

For the papers included in this systematic review, the intervention group had to follow a rehabilitation program using BFRT to improve their muscle strength, isolated or combined with other therapeutic strategies, in any dosage and provided in any setting (inpatient, outpatient or home treatment).

### 2.6. Outcome Measures

All the analyzed motor control outcomes, including muscle strength, mobility, tone, coordination and dexterity in the upper and/or lower limbs, were registered.

### 2.7. Data Extraction and Analysis

The search results were uploaded to the bibliographical citation manager Zotero 7 and duplicates were removed. After, two independent reviewers completed an Excel 365 template with the title and abstract of all the uploaded citations to be analyzed. The articles selected were obtained in full text and were evaluated by the research team to determine their relevance to the review’s aims.

The following data were extracted from the papers: the study design, EDSS and type of EM, intervention protocol, sample, dosage, outcome measures or data collection procedure and results. The authors independently collected these data following the CONSORT 2010 statement [21] when possible and eventually reached a consensus on the extracted data, resolving disagreements through discussion.

### 2.8. Assessment of the Methodological Quality of the Studies and Risk of Bias

Generally, systematic reviews have focused exclusively on randomized clinical trials (RCTs), thus discarding observational studies due in part to difficulties in their evaluation of methodological quality. However, in many areas of healthcare, there is a lack of high-quality RCTs, and to guarantee the critical and objective evaluation of studies not included in this category, proven tools are available, such as the Quality Index of Downs and Black [22]. Although it is true that the designs of RCTs, cohort or case–control studies present fundamental differences, in all of them, it is necessary to analyze the characteristics of the intervention, the confounding factors and the results. In the present systematic review, considering the type of studies included, the Quality Index of Downs and Black tool was used to evaluate their methodological quality. The tool consists of 27 items that assess the following: the content quality (10 items), external validity (3 items), internal validity and bias (7 items), confounding factors (6 items) and weighting statistical power (1 item). Higher scores indicate higher methodological quality, with a maximum possible score of 27 points.

Additionally, the articles were classified according to the levels of evidence and grades of recommendation for diagnosis studies established by the Oxford Center for Evidence-Based Medicine [23].

The Cochrane Handbook for Systematic Reviews of Interventions [24] was used by two the independent reviewers to assess the risk of bias, assessing six different domains:

- Selection bias: relates to the recruiting process and participant allocation. To analyze it, randomization and allocation concealing must be considered.
- Performance bias: refers to systematic differences between groups in the care that is provided, or exposure to factors other than the interventions of interest. To analyze it, blinding procedures must be examined.
- Detection bias: refers to systematic differences between groups in how outcomes are determined and may occur during the intervention and follow-up. The blinding of outcome assessors must be considered when analyzing it, since it may reduce the risk of bias.
- Attrition bias: refers to systematic differences between groups in withdrawals from a study. It occurs when there are withdrawals that lead to incomplete outcome data or when withdrawals in both groups differ significantly.
- Reporting bias: refers to systematic differences between reported and unreported findings. This can occur once the study is finished and it is due to the selective reporting of results, reporting only statistically significant data.
- Other biases: occur when reviewers include methodological aspects that are not assessed in the domains described before. They relate mainly to certain trial designs, such as crossover trials.

Each study was assessed independently and was considered to have a “low risk of bias” when each domain was addressed properly. Otherwise, it was considered to have a “high risk of bias”. If a study did not provide enough information, it was considered “dubious”. Disagreement was resolved through discussion with a third reviewer. The RoB 2 tool was used to obtain the relevant diagrams [25].

## 3. Results

**Figure 1:**
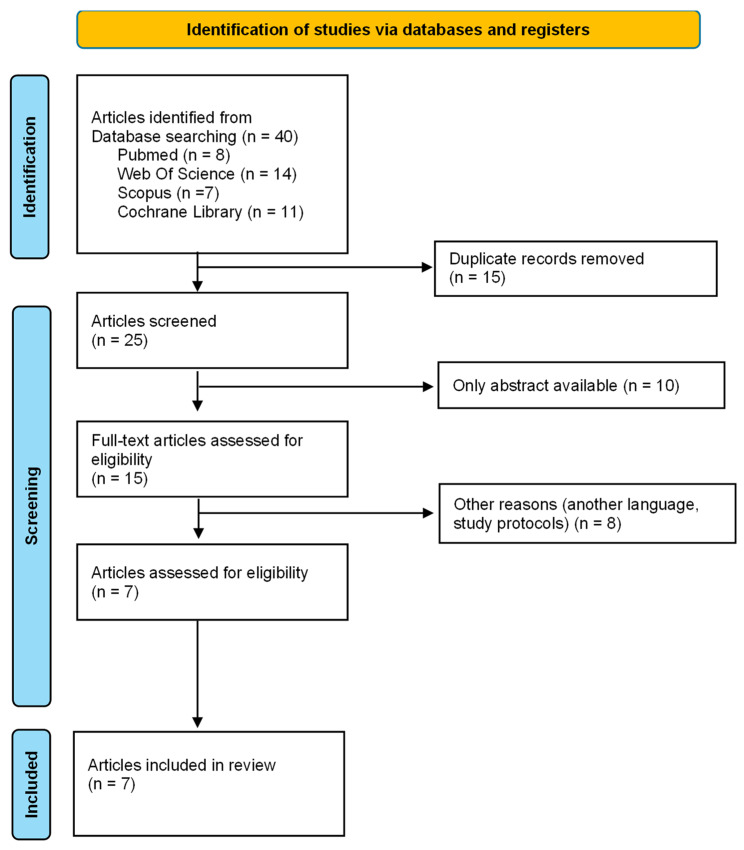
PRISMA Flow chart for identifying studies for systematic review.

The identification of the articles in the different databases and their selection process are detailed in Figure 1. The initial search found 40 articles. After eliminating duplicates and those that did not meet the inclusion criteria, a total of seven articles were obtained in the present systematic review. The characteristics of the articles are shown in Table 3. 

### 3.1. Sample Characteristics

The data provided by the seven studies derive the following general information: A total of 71 subjects received treatment through the BFRT system, both men and women. The studies presented a range of subjects, from one to twenty. The EDSS scores were from 0 to 7 points; it was not possible to highlight a median due to the absence of data from some studies; the forms of the presentation of the disease were both RR and SP or PP. However, it was impossible to know the total number of subjects presenting each of them. The sample presented a mean age of 52. 9 (±24.20) years but had an unknown mean number of years of disease evolution due to the absence of data in different investigations. However, from the data obtained, we can point out a range from less than 1 year to 33 years of disease evolution.

Cohen et al. [26] developed a case study of a 54-year-old woman with PP MS with 13 years of evolution, with an EDSS score of 3.0. Darvishi et al. [27] presented two intervention groups exposed to the BFRT system, but data were only obtained from one of the groups with 20 subjects aged 47.11 (±16.25). However, the different forms of disease presentation and years of disease evolution were not shown. The study by Lamberti et al. [28] presented eleven subjects with PP MS and an age of 54 (±11.6) years and five persons with SP MS with 14 (±9) years of disease evolution. Freitas et al. [29] presented 15 subjects of 45.7 (±9.4) years of age with the RR type of MS. The years of disease evolution were not shown. The study by Mañago et al. [30] included fourteen subjects aged 55.4 (±6.2) years, with five with SP MS, three with PP MS and six with RR MS, with a median EDSS score of 6.5 and a range of disease evolution from 3 to 33 years. Brown et al. [31] presented one woman with RR MS aged 30 years and with an EDSS score of 5.5, with less than one year of disease evolution. Finally, the study by Hill [32] presented sixteen subjects, with fourteen women aged 41 (±12) years and two men aged 45 (±8) years with RR MS. The years of disease evolution in this study were unknown. The median EDSS were not shown in several articles [27,28,29,32].

### 3.2. Intervention Characteristics

All the studies presented at least one protocol that applied the BFRT program, with five studies including the BFRT system in addition to a LL-RT exercise and two aerobic exercise articles, one with treadmill walking and the other with a cycloergometer (Table 3).

#### 3.2.1. BFRT Combined with Strengthening Exercises

A total of five articles [26,29,30,31,32] applied a muscle strengthening program through LL-RT in combination with BFRT. The mean number of weeks of intervention in the protocols was 8.2 weeks. The article that presented the least presented 1 week of intervention [29], and the studies that presented the most weeks of intervention presented 12 weeks [26,32]. The mean duration of the interventions could not be determined since none of the protocols referred to the data of this intervention modality.

Of the five articles that analyzed LL-RT in combination with BFRT, only two presented a control group [29,32]. The experimental group of Freitas et al. [29] performed a LL-RT protocol (20%-1RM) in combination with a BFRT of two exercises (leg-press and knee extension), with four series with 30/15/15/15 repetitions, with a rest of 1 min between the series and 3 min between the exercises, while the control group performed an HL-RT protocol (70%-1RM) with the same exercises, series and rests, but with a range of 8–10 repetitions per series. The experimental group of Hill et al. [32] performed the same scheme of sets and repetitions but executed a greater number of exercises (chest-press, seated-rows, shoulder-press, leg curl, leg-extension and leg-press). Their control group performed an HL-RT protocol (65% of 1RM) with the same scheme of exercises, sets and rests as the experimental protocol but applied a repetition range of 8–12 repetitions. Both protocols performed two sessions for 12 weeks. The other investigations only analyzed the effect of LL-RT in combination with BFRT in people with MS, without making any comparison with another control group.

The strengthening exercises selected were mostly monoarticular, such as the gluteal bridge, hip abduction, knee extension, heel raise, hamstring curl, knee extension [26,29,30,31,32]; although, three studies [29,31,32] also showed multiarticular exercises such as the squat or leg press. Likewise, the inclusion of upper limb and trunk exercises such as the chest-press, seated-rows and shoulder-press also stands out [32].

Of the five investigations of LL-RT in combination with BFRT [26,29,30,31,32], four presented a multi-week protocol; the protocols were 8 weeks in duration in the articles by Mañago et al. [30] and Brown et al. [31], whereas 12-week protocols were presented in the articles by Cohen et al. [26] and Hill et al. [32]. On the other hand, Freitas et al. [29] developed one single training session in their entire study. In the four investigations that presented several weeks of intervention [26,30,31,32], two sessions per week were performed; in four studies [26,29,31,32], a percentage of the arterial occlusion pressure (AOP) was used, and in one study [30], the limb occlusion pressure (LOP) was employed, with each investigation presenting a different initial dose and progression. Cohen et al. [26] applied an AOP of 50% during their first four sessions, 60% from the fifth to the eighth intervention session and from the twelfth to the seventeenth, 65% from the ninth to the eleventh and 70% from the nineteenth to the twenty-first. Freitas et al. [29] applied an AOP of 50% during their whole protocol. Mañago et al. [30] applied an LOP of 60 to 80%, but they did not present the data by intervention. Brown et al. [31] applied an AOP of 70% during the first week of their intervention, which increased weekly by 2% until it reached a maximum of 80%. Hill et al. [32] applied an AOP of 60% continuously during the protocol. In four articles [29,30,31,32], the cuff was deflated during the rest between exercises but not during the rest between sets of the same exercise. Likewise, the rest between the exercises and between the sets differed in time from each other. One of the papers [26] did not determine whether the cuff pressure was deflated at any time. In five of the articles [26,30,31,32], five sets of 30, 15, 15, 15 and 15 repetitions were performed for each exercise.

#### 3.2.2. BFRT Combined with Aerobic Exercise

Two studies investigated aerobic exercise in combination with the BFRT system [27,28]. The mean number of weeks of intervention was 7 weeks, with 8 weeks in the protocol with the most weeks and 6 weeks in the protocol with the least number of weeks. The frequency of intervention was two sessions per week in both protocols. The mean intervention time between both studies was 52’5 min, with 45 min in the protocol with the least amount and 60 min in the protocol with the most amount.

Both investigations [27,28] included a control group and an experimental group. In the case of Darvisi et al. [27], four study groups were defined: a control group that did not perform any specific activity, only their daily routine; an experimental group that performed aerobic exercise without combining it with BFRT; a second experimental group that performed aerobic exercise in combination with BFRT; and a fourth experimental group that was only exposed to the BFRT system, without determining what type of intervention, exercise or activity was performed. Lamberti et al. [28] presented two groups, an experimental group that performed aerobic exercise in combination with BFRT and a control group that performed the same protocol as the previous group except for the use of BFRT.

The two studies performed two sessions per week for a period of 8 and 6 weeks, respectively. In both studies, the experimental protocol began with a warm-up phase and ended with a cool-down phase. However, Lamberti et al. [28] also included the active and passive stretching of the musculature involved. The total time applied in the research by Darvishi et al. [27] was 45 min, while Lamberti et al. [28] used 60 min. In the first protocol, a pressure of 150–160 mmHg was applied continuously, and in the second protocol, a pressure of 30% of the systolic blood pressure (SBP) was applied. In the research conducted by Darvishi et al. [27], an intensity corresponding to 50–60% of the maximum heart rate of each patient was reached but the exact amount of time during which it was applied was not detailed, while the protocol of Lamberti et al. [28] included five series of walking at a low speed (60 steps per minute) marked by a metronome; each series had three sequences of 1 min of activity and 1 min of rest (sitting on a chair). Once a series was completed, a 3 min rest was developed during which the cuff pressure was reduced to 0 mmHg.

### 3.3. Outcome Measures

Freitas et al. [29] and Hill et al. [32] measured issues concerning the RPE, pain and training volume. The research by Freitas et al. [29] showed that the RPE achieved during the high-load exercise was significantly higher (*p* ≤ 0.01) than for the low-load exercise and BFRT. Hill et al. [32] showed that there were no significant differences in the RPE generation between both interventions (*p* = 0.132 to >0.999). With respect to the pain measured in the study by Freitas et al. [29], statistically significant differences (*p* < 0.05) were observed for the LLT-BFR protocol versus the HL-RT in the measurement immediately prior to the start of each series for the experimental protocol. However, the pain measured immediately after each series showed no significant differences (*p* > 0.05) between the protocols, except for the knee extension which showed a statistically significant result (*p* < 0.05) in the experimental group. In parallel, the effect of the BFRT system does not seem to have greater effects on delayed onset muscle soreness (DOMS), stiffness and physical fatigue (*p* > 0.05) than a conventional protocol. On the other hand, the analysis performed on the training volume (“normalized load” and “tonnage”) show no difference between the two protocols, except for some movements that showed a faster adaptation in favor of the experimental protocol. In the case report by Cohen et al. [26], two of the three outcome measures (the twelve-item MS walking scale (MSWS-12) and the Fatigue Severity Scale (FSS)) came close to reaching the minimum detectable change (CMD) of the test, while this was reached in the patient-specific functional scale (PSFS). In the case report by Brown et al. [31], the CMD was obtained for the Berg balance scale (BBS) and the activity-specific balance confidence (ABC) scale, as well as for the five-times sit-to-stand (FTSTS) test, which showed an improvement in the strength and functional capacity and strength measured through isometric dynamometry for all the movements assessed. However, the MFIS did not achieve the CMD.

The prospective cohort carried out by Darvishi et al. [27] showed favorable results for the experimental protocols (aerobic exercise, BFRT and the combination of both), which showed better results in balance, strength and thigh hypertrophy than the control group that did not perform any activity, highlighting the fact that the group performing BFRT in combination with aerobic exercise was shown to be inferior to the other two experimental protocols (aerobic exercise in isolation and the BFRT program in isolation). Mañago et al. [30] conducted a prospective cohort that yielded positive results in all the domains of the plantar flexor, hip abductor and knee extensor muscle strength of both the more and less affected sides, improving from 12% to 28% over the baseline values. The Berg balance scale reached the CMD, as did the fatigue measured through the PSFS questionnaire. The modified fatigue impact scale (MFIS), MSWS-12, 30 s chair–stand test (30STS) and timed 25-foot walk test (T25FW) tests showed improvements in their score but did not reach the CMD.

The study by Lamberti et al. [28] showed that the BFRT system in combination with aerobic exercise presented a shorter distance and total walking time in the intervention than the control group, as well as a lower heart rate during the exercise and a lower RPE. Regarding the walking speed, both groups presented a significant difference at the end of the protocol in the time–treatment factor (*p* = 0.003), with all the subjects improving their baseline condition and maintaining the results in the follow-up assessment in the experimental group but not in the control group. In turn, the experimental group showed a statistically significant difference at the intergroup level (*p* = 0.001).

It should be noted that out of the seven studies, side effects occurred in only one study, which was a muscle strain in only one subject [30]. No other adverse effects were recorded in the rest of the included studies.

### 3.4. Assessment of the Methodological Quality of the Studies and Risk of Bias

The score obtained on the Downs and Black scale reflects a heterogeneous methodological quality, finding a mean of 15.28 points out of a total of 27; the articles that showed the lowest methodological quality were those by Cohen et al. [26] and Brown et al. [31], with scores of 11 out of 27, while the study that obtained the highest methodological quality was that of Lamberti et al. [28], with a score of 23 out of 27. The results obtained on the scale are shown in Table 4.

Regarding the level of evidence, most of the articles presented a low level of evidence and grade of recommendation: three studies presented a 4C grade, two articles presented a 3B grade, one article presented a 1C grade and, finally, one article presented a 1B grade. The results obtained are shown in Table 5.

**Figure 2:**
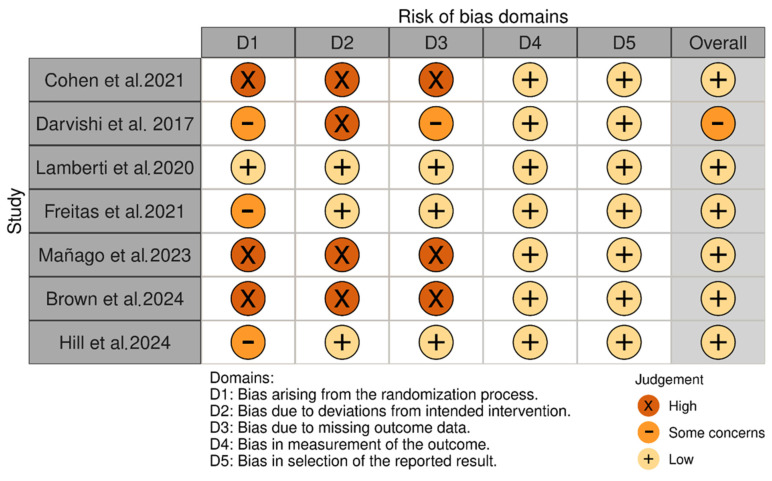
Risk of bias analysis [26,27,28,29,30,31,32].

**Figure 3:**
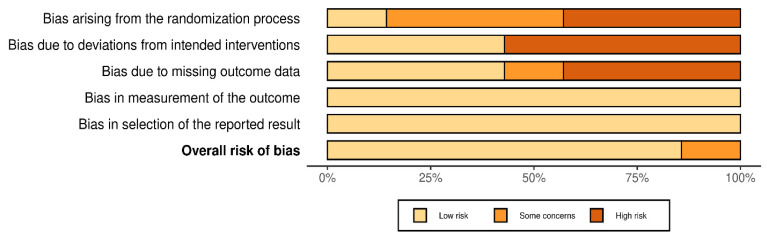
Summary plot of risk of bias.

The risk of bias of the included studies was moderate, with a higher risk in the first items, highlighting the following: selection bias, due to the absence of a control group in some studies, as well as allocation concealment in others; performance bias, again affected by the absence of control groups or, failing this, by no presentation of an adequate adjustment for confounding factors; and detection bias, with a high risk due to the absence of a control group and an inadequate description of the intervention protocol in one of the studies [27]. The analysis of the risk of bias is presented in detail in Figure 2 and Figure 3.

## 4. Discussion

To our knowledge, this is the first systematic review about the clinical effects and feasibility of BFRT in people with MS. In the past 20 years, the interest in the clinical effect of physical exercise in people with MS has increased, leading to a multitude of studies showing its positive effects on fatigue [33], mobility and balance [34], social participation [35] and quality of life [36], consolidating it as a safe, effective and low-cost intervention [37] in the rehabilitation of people with MS.

The findings in this work indicate positive changes in several outcome measures, such as the maximum isometric strength [26,27,30,31] and general strength of the lower limbs in the 30STS test [30]; the muscular hypertrophy of the lower limbs [27] in the fatigue perceived [26,30,31]; gait speed [28] in the MSW-12 [26,30], 10MWT and T25FW scale [30]; and balance [27,30] in the pain during and after exercise and RPE [29,32]. However, not all papers point in this direction [28] in terms of fatigue, balance, and quality of life compared to control groups. In turn, it should be noted that only one subject reported adverse effects derived from the experimental protocol, this being muscle strain in the hip adductor musculature [30]. However, it was only necessary to modify the workload applied during the protocol, and no further adverse effects were reported.

Nevertheless, we must highlight that the experimental groups presented great heterogeneity in the way vascular compression was determined, with up to three different ways (AOP, LOP and SBP) of measuring it (although two of them were the same measurement with different nomenclature) and with differences in the magnitude and progression of vascular pressure during the protocol.

Compared to the results indicated above, prior systematic reviews about BFRT in other neurological disorders indicated that LL-RT plus BFRT appears to represent a safe and effective training modality for producing increases in maximal muscle thickness, strength and functional capacity, respectively, in persons with neurological disorders. However, LL-RT plus BFRT should not be uncritically administered to all individuals with neurological disorders. Extra caution is recommended when patients are easily fatigued or otherwise presenting known risk factors for experiencing adverse events with BFRT. On the other hand, Vinolo-Gil et al. [18] conducted a systematic review about the effects of the BFRT system in people with other neurological pathologies to MS, finding that all the outcome measures obtained positive results except for balance. The findings of our research are in line with this, but two studies [27,30] in our work presented positive effects on balance outcomes, possibly due to the different protocols used, as well as the dosage of the aerobic treatment combined with BFRT. Other systematic reviews with meta-analysis have been conducted by Lixandrao et al. [38] and Grønfeldt et al. [39] about the effects of BFRT in healthy subjects. The review by Lixandrao et al. [38] analyzed data from 12 investigations that studied the effects of a conventional training protocol versus LL-RT in combination with BFRT, including a total of 460 people. The results of the review indicated that a conventional training protocol can induce around 7.36% more gains in terms of strength than a LL-RT protocol with BFRT. However, Grønfeldt et al. [39] included a total of 16 studies with a total of 310 people. Their results showed that there were no differences in terms of strength or muscle mass for any of the groups. So, the conditions of clinical effectiveness could be due to the basal strength levels of the samples or the protocols used.

Considering the data presented in our systematic review, BFRT could be considered a strategy with therapeutic potential for people with MS, since it appears to be as effective a tool as conventional training models in healthy subjects. Furthermore, different investigations presented improvements with respect to all the outcome measures relevant to this type of population, with particular interest in the investigations conducted by Lamberti et al. [28], Freitas et al. [29] and Hill et al. [32], which showed that BFRT generated the same results as a conventional protocol but at a lower degree of RPE; so, for subjects who present a high level of fatigue and reluctance to undertake exercise, it might be a tool of choice. On the other hand, the versatility of the tool stands out, since by being able to combine it with aerobic exercises and obtaining results in terms of strength [27], it could allow for the training of motor deficits through task-focused training while achieving strength effects.

It should be noted that BFRT has proven to be effective in other populations such as older people. Centner et al. [40] conducted a systematic review with meta-analysis that revealed that, although conventional training showed better results for improvement in strength and hypertrophy than a protocol based on LL-RT and BFRT, BFRT showed superior improvements in the strength of the lower limbs compared to a conventional walking protocol versus the same protocol in combination with BFRT. On the other hand, despite the findings described, Zhang XZ et al. [41] described that a LL–RT protocol in combination with BFRT could achieve the same effects as a conventional protocol but in less time in older people with sarcopenia and thus is a more efficient strategy for people with frailty. All this could reinforce the hypothesis of using this training system in populations with MS, in which the levels of muscle strength and fragility could be present from the early stages of and/or throughout the disability process.

Future publications should present higher methodological quality, including the following recommendations: (a) a control group with subjects with MS with the adequate control of confounding factors (age, gender, years of disease evolution and the MS forms of presentation); (b) the adequate randomization and blinding of the professionals in charge of performing the outcome measures; (c) an adequate sample size; (d) to control and describe the diet of study participants or their dietary intake (including chemicals and food additives), especially considering that the myelin sheath is compromised since none of the papers included in this systematic review address this topic; (e) to establish new lines of work that combine the performance of BFRT and study its effects on the testosterone and muscle mass of people with MS; (f) to know the effects of other techniques complementary to BFRT or alternatives that could have an impact on blood flow as a hyperbaric oxygen therapy for MS.

There are different limitations in this review that should be highlighted. Given the heterogeneity of the outcome measures in the different types of intervention and the different forms of vascular occlusion, it was impossible to develop a meta-analysis. Both the methodological quality of the studies and the risk of bias were moderate, so the conclusions of the different studies should be taken with caution, particularly those that do not present control groups [26,30,31]. Other limitations that could be highlighted are the low level of recommendation of some of the studies and the low sample size of most of the studies. Finally, the absence of data on the EDSS score and the forms of the presentation of the disease, as well as the years of the evolution of the disease, do not allow us to extrapolate the results of the investigations to all subjects with MS.

## 5. Conclusions

BFRT in people with MS appears to be an effective, safe and versatile strategy for rehabilitation aims. It seems to be more effective in improving gait speed than conventional rehabilitation protocols and to show positive clinical effects on strength, hypertrophy and balance outcomes. Nevertheless, future research should be conducted with better methodological quality to ensure the potential benefits of BFRT in people with MS, since the studies presently analyzed have a high risk of bias and methodological limitations.

## Figures and Tables

**Table 1:** Search filters in databases.

Database	Search Filter
*PubMed*	Text availability: full text. Article Type: all. Publication date: without restriction.
*Web of Science*	Text availability: all. Article Type: all. Publication date: without restriction.
*Scopus*	Year: without restriction. Document type: article. Language: English. Open access: all open access.
*Cochrane Library*	Years: without restriction. Language: English.

**Table 2:** Keywords and equations for searching.

Database	Search Equation
*PubMed*	((“blood flow restriction” [Title/Abstract]) OR (“blood flow restricted” [Title/Abstract]) OR (“blood flow restriction exercise”[Title/Abstract]) OR (“blood flow restriction training”[Title/Abstract]) OR (“blood flow restriction therapy”[Title/Abstract]) OR (“occlusion training”[Title/Abstract]) OR (kaatsu[Title/Abstract])) AND ((“rehabilitation”[Title/Abstract]) OR (“muscle strength”[Title/Abstract]) OR (“upper limb”[Title/Abstract]) OR (“lower limb”[Title/Abstract]) OR (“resistance training” [Title/Abstract])) AND (“multiple sclerosis” [Title/Abstract])
*Scopus*	((“blood flow restriction” [Title/Abstract]) OR (“blood flow restricted” [Title/Abstract]) OR (“blood flow restriction exercise”[Title/Abstract]) OR (“blood flow restriction training”[Title/Abstract]) OR (“blood flow restriction therapy”[Title/Abstract]) OR (“occlusion training”[Title/Abstract]) OR (kaatsu[Title/Abstract])) AND ((“rehabilitation”[Title/Abstract]) OR (“muscle strength”[Title/Abstract]) OR (“upper limb”[Title/Abstract]) OR (“lower limb”[Title/Abstract]) OR (“resistance training” [Title/Abstract])) AND (“multiple sclerosis” [Title/Abstract])
*Web of Science*	TS = (((“blood flow restriction”) OR (“blood flow restricted”) OR (“blood flow restriction exercise”) OR (“blood flow restriction training”) OR (“blood flow restriction therapy”) OR (“occlusion training”) OR “kaatsu”) AND ((“rehabilitation”) OR (“muscle strength”) OR (“upper limb”) OR (“lower limb”) OR (“resistance training”)) AND (“multiple sclerosis”))
*Cochrane Library*	Title, abstract and keywords: ((“blood flow restriction”) OR (“blood flow restricted”) OR (“blood flow restriction exercise”) OR (“blood flow restriction training”) OR (“blood flow restriction therapy”) OR (“occlusion training”) OR (“kaatsu”)) AND ((“rehabilitation”) OR (“muscle strength”) OR (“upper limb”) OR (“lower limb”) OR (“resistance training”))

**Table 3:** Characteristics of the articles.

Author	Study Design	EDSS	Type of MS	Intervention	Sample (n)	Age (Years)	Time Since Diagnosis (Years)	Dosage for Experimental Protocols	Outcome Measure	Results
*Cohen et al.* [26]	Case report.	3.0	PP	Squat. Standing hip flexion. Dorsiflexion. Standing hamstring curl.	1	54	13	**Interventions**: 2 days per week for 12 weeks. **Sets and repetitions**: 30/15/15/15 or until failure concentric. **AOP**: 50% 1° to 4° session. 60% 5° to 8° session and 12° to 17°. 65% 9° to 11° session and 18° session. 70% 19° to 21° session.	MSWS-12. FSS. PSFS. Handheld dynamometry.	**MSWS-12**: Improvement of 20.4 points at 6 weeks and 19 points at 12 weeks. **FSS**: Unchanged at 6 weeks but improvement of 1.8 points at 12 weeks. **PSFS**: Improvement of 6 points at 6 and 12 weeks. **Handheld dynamometry**: Improvement in more affected and less affected limb.
*Darvishi et al.* [27]	Semi-experimental pre-test–post-test designed research.	2.8 ± 1.2	NA	Aerobic training with ergometer.	40	45.31 ± 11.4	NA	**Interventions**: 2 days per week for 8 weeks. **Duration**: 45 min. **Intensity**: 50–60% maximum heart rate. **AOP**: 150–160 mmHg	BBS. MIS. Thigh hypertrophy.	**BBS**: Significant changes in every group except control group. **MIS**: Significant changes in every group except control group. **Thigh hipertrophy**: Significant changes in every group except control group.
*Lamberti et al.* [28]	Pilot randomized controlled, parallel-group clinical trial.	6.1 ± 0.2	PP, SP	5 bouts of walking at 60 steps/min.	11	54 ± 11	14 ± 9	**Interventions**: 2 days per week for 6 weeks. 1 set of 1 min followed by rest of 1 min, 3 times. After 1 bout of exercise, rest was performed, with 1 set of rest for 1 min without oclussion. Walking speed increased weekly by 3 steps/minute when previous level was tolerated. **Duration**: 1 h. **AOP**: 30% SBP during the set. 00 mmHg during the break inter-series.	T25FW. 6-MWT. BBS. FSS. MFIS. SF-36. MSIS-29.	**Gait speed**: Significant improvement in both groups and significant difference between groups in favor of experimental group. No improvements were found in any other tests.
*Freitas et al.* [29]	Randomized crossover trial.	1.87 ± 1.51	RR.	Leg press. Knee extension.	8	45.7 ± 9.4	NA	**Interventions**: 4 sets of 30–15–15–15 repetitions performed at 20% of 1RM, with 1 min rest interval between sets and 3 min between exercises. **AOP**: 50% during set. 0% during 3 min rest interval.	RPE. DOMS. Pain.	**RPE**: Control group elicited significantly greater overall RPE response than experimental group. **Pain**: Pre-intervention pain was significantly greater in experimental group. Both groups showed same pain in post-intervention measurement.
*Mañago et al.* [30]	Prospective cohort study.	6.0–7.0	RR, PP, SP.	Leg press. Calf press. Hip abduction.	14	55.4 ± 6.2	3–33	**Interventions**: Twice weekly for 8 weeks. 4 sets of 30–15–15–15 repetitions performed at 20% to 30% of 1RM. **LOP**: 60% to 80% of maximal LOP during set. 0% during rest between exercise	Handheld dynamometry. 30STS. BBS. T25FW. MFIS. MSW-12. PFSF. Daily step count.	**Handheld dynamometry**: Improvement in more affected and less affected limb. **30STS**: Improvement of 1.9 repetitions. **BBS**: Improvement of 5.3 points. **MFIS**: Improvement of 8.8 points. **T25FW**: Improvement of 3.3 s. **MSW-12**: Improvement of 3.6 points. **PFSF**: Improvement of 2.9 points. **Daily step count**: Improvement of 333 steps.
*Brown et al.* [31]	Case report.	5.5	RR	Recumbent stepper. Straight leg raise. Gluteal bridge. Tall kneeling heel-taps.	1	30	<1 year	**Interventions**: 2 days per week for 8 weeks. 10 min of aerobic warm-up of at least 100 steps per minute, Borg 3/10–4/10. **Sets and repetitions**: 30, 15, 15, 15. **AOP**: 70% for first week. 2% increase every week until meeting 80% 0% during rest between exercise (1’) but not during rest between sets (30’’)	ABC. BBS. FTSTS. 10MWT. MFIS. Handheld dynamometry.	**ABC**: Improvement of 31.9%. **BBS**: Improvement of 9 points. **FTSTS**: Improvement of 4.66 s **10MWT**: Improvement of 0.11 points. **MFIS**: Improvement of 15 points. **Handheld dynamometry**: Improvement in more affected and less affected limb.
*Hill et al.* [32]		0–5.5	RR.	Chest-press. Seated-rows. Shoulder-press. Leg curl. Leg extension. Leg-press.	15	Women 41 ± 12 Men: 45 ± 8	NA	**Intervention**: Twice weekly for 12 weeks. **Sets and repetitions**: 75 repetitions over 4 sets (1 × 30, 3 × 15), 30% of estimated 1RM. **AOP**: 60% during set. 0% between exercises.	RPE. Exercise load Tonnage.	Better normalized load in early phase in experimental group for chest-press, seated-rows and leg curl. Equal normalized load for shoulder-press, leg extension and leg press. There were no significant differences in RPE between groups except for shoulder-press at week 1, favoring the experimental group.

Arterial occlusion pressure (AOP); Berg balance scale (BBS); delayed-onset muscle soreness (DOMS); fatigue severity scale (FSS); limb occlusion pressure (LOP); maximum isometric strength (MIS); modified fatigue impact scale (MFIS); multiple sclerosis impact scale–29 (MSIS-29); patient-specific functional scale (PSFS); rating of perceived exertion (RPE); systolic blood pressure (SBP); timed 25-foot walk test (T25FW); 6 min walking test (6-MWT); 12-item multiple sclerosis walking scale (MSWS-12); 36-item short-form health survey (SF-36).

**Table 4:** Quality Index of Downs and Black.

	Cohen et al. [26]	Darvishi et al. [27]	Lambert et al. [28]	Freitas et al. [29]	Magañano et al. [30]	Brown et al. [31]	Hill et al. [32]
**REPORTING**							
Is the hypothesis/aim/objective of the study clearly described?	Yes	Yes	Yes	Yes	Yes	Yes	Yes
Are the main outcomes to be measured clearly described in the Introduction or Methods section?	Yes	Yes	Yes	Yes	Yes	Yes	Yes
Are the characteristics of the patients included in the study clearly described?	Yes	No	Yes	Yes	Yes	Yes	Yes
Are the interventions of interest clearly described?	Yes	No	Yes	Yes	Yes	Yes	Yes
Are the distributions of principal confounders in each group of subjects to be compared clearly described?	No	No	No	No	No	No	No
Are the main findings of the study clearly described?	Yes	Yes	Yes	Yes	Yes	Yes	Yes
Does the study provide estimates of the random variability in the data for the main outcomes?	No	Yes	Yes	Yes	Yes	No	Yes
Have all important adverse events that may be a consequence of the intervention been reported?	No	No	Yes	Yes	Yes	No	No
Have the characteristics of patients lost to follow-up been described?	Yes	Yes	Yes	Yes	Yes	Yes	Yes
Have actual probability values been reported (e.g., 0.035 rather than <0.05) for the main outcomes except where the probability value is less than 0.001?	No	Yes	Yes	No	Yes	No	Yes
**EXTERNAL VALIDITY**							
Were the subjects asked to participate in the study representative of the entire population from which they were recruited?	Unable to determine	Unable to determine	Yes	Unable to determine	Yes	Unable to determine	Unable to determine
Were those subjects who were prepared to participate representative of the entire population from which they were recruited?	No	No	No	No	No	No	No
Were the staff, places and facilities where the patients were treated representative of the treatment the majority of patients receive?	Yes	Yes	Yes	No	No	Yes	No
**INTERNAL VALIDITY-BIAS**							
Was an attempt made to blind study subjects to the intervention they have received?	No	No	No	No	No	No	No
Was an attempt made to blind those measuring the main outcomes of the intervention?	No	No	Yes	No	No	No	No
If any of the results of the study were based on “data dredging”, was this made clear?	Yes	Yes	Yes	Yes	Yes	Yes	Yes
In trials and cohort studies, do the analyses adjust for different lengths of follow-up of patients, or in case–control studies, is the time period between the intervention and outcome the same for cases and controls?	No	Yes	Yes	Yes	No	No	Yes
Were the statistical tests used to assess the main outcomes appropriate?	No	Yes	Yes	Yes	Yes	No	Yes
Was compliance with the intervention/s reliable?	Yes	Yes	Yes	Yes	Yes	Yes	Yes
Were the main outcome measures used accurate (valid and reliable)?	Yes	Yes	Yes	No	Yes	No	Yes
Were the patients in different intervention groups (trials and cohort studies) or were the cases and controls (case–control studies) recruited from the same population?	Unable to determine	Yes	Yes	Unable to determine	No	Unable to determine	Unable to determine
Were study subjects in diferent intervention groups (trials and cohort studies) or were the cases and controls (case–control studies) recruited over the same period of time?	Unable to determine	Yes	Yes	Unable to determine	No	Unable to determine	Unable to determine
Were study subjects randomized to intervention groups?	No	Yes	Yes	Yes	No	No	Yes
Was the randomized intervention assignment concealed from both patients and health care staff until recruitment was complete and irrevocable?	No	Yes	Yes	Yes	No	No	Unable to determine
Was there adequate adjustment for confounding in the analyses from which the main findings were drawn?	No	No	No	No	No	No	No
Were losses of patients to follow-up taken into account?	Yes	Yes	Yes	Yes	Yes	Yes	Yes
Did the study have sufficient power to detect a clinically important effect where the probability value for a difference being due to chance is less than 5%?	No	No	Yes	No	No	No	No
**Total Score**	11/27	17/27	23/27	15/27	15/27	11/27	15/27

**Table 5:** Levels of evidence and grades of recommendation.

Cohen et al. [26]	Darvishi et al. [27]	Lambert et al. [28]	Freitas et al. [29]	Magañano et al. [30]	Brown et al. [31]	Hill et al. [32]
4C	3B	1B	3B	4C	4C	1C
